# Treatment outcomes of fruquintinib combined with PD-1 inhibitors in advanced colorectal cancer: a reconstructed patient-level meta-analysis of retrospective real-world studies

**DOI:** 10.1186/s12885-026-16420-5

**Published:** 2026-07-15

**Authors:** Yan Liu, Jing-Ting He, Guo-He Lin, Chen Fu, Ru-Bo Cao, Bi-Cheng Wang

**Affiliations:** 1 Department of Oncology, Sinopharm Dongfeng General Hospital, Hubei University of Medicine, Shiyan, Hubei 442008 PR China; 2 Department of Oncology, the Second Affiliated Hospital of Anhui Medical University, Hefei, 230601 China; 3 Department of Dermatology, Traditional Chinese and Western Medicine Hospital of Wuhan, Tongji Medical college, Huazhong University of Science and Technology, Wuhan, Hubei 430022 China; 4 Cancer Center, Union Hospital, Tongji Medical College, Huazhong University of Science and Technology, 1277 Jiefang Avenue, Wuhan, 430022 China

**Keywords:** Advanced colorectal cancer, Fruquintinib, PD-1 inhibitors, Real-world studies, Patient-level survival analysis

## Abstract

**Background:**

Fruquintinib is an established treatment option for patients with advanced colorectal cancer (CRC) who have progressed after standard therapies, including oxaliplatin- and irinotecan-based chemotherapy. Although this population generally shows limited sensitivity to anti–PD-1/PD-L1 immunotherapy, the combination of fruquintinib and PD-1 inhibitors has been widely used in clinical practice. In this reconstructed patient-level meta-analysis, we aimed to characterize survival outcomes reported in retrospective real-world studies evaluating fruquintinib combined with PD-1 inhibitors.

**Methods:**

Public databases, including PubMed, Web of Science, Embase, and the Cochrane Central Register of Controlled Trials (CENTRAL), were systematically searched on January 26, 2026. Patient-level survival data were reconstructed from published Kaplan–Meier curves using the “IPDfromKM” package in R. The “survival” package was used to analyze reconstructed overall survival (OS) and progression-free survival (PFS). The “meta” package was used to pool objective response rate (ORR) and disease control rate (DCR).

**Results:**

A total of 10 retrospective studies involving 609 patients were included, all conducted in China. The reported median OS ranged from 14.9 to 19.5 months, and the pooled median OS was 15.7 months. The reported median PFS ranged from 3.8 to 7.0 months, and the pooled median PFS was 5.5 months. The pooled ORR was 12% (95% CI, 7–18), and the pooled DCR was 74% (95% CI, 68–81). No significant publication bias was detected in the analyses of response rates.

**Conclusion:**

This reconstructed patient-level meta-analysis provides a descriptive summary of reported outcomes associated with fruquintinib combined with PD-1 inhibitors in patients with advanced CRC across retrospective real-world studies. However, safety outcomes were not systematically reported and therefore could not be comprehensively evaluated. Given the absence of a comparator group, the current evidence does not allow assessment of whether the addition of PD-1 inhibitors improves outcomes compared with fruquintinib monotherapy. Prospective randomized controlled trials are warranted to further characterize the clinical outcomes and safety profile of this treatment strategy.

**Supplementary Information:**

The online version contains supplementary material available at https://doi.org/10.1186/s12885-026-16420-5.

## Introduction

Fruquintinib is currently a standard treatment option for patients with advanced colorectal cancer (CRC) who have previously received oxaliplatin- and irinotecan-based chemotherapy [1]. In the phase III FRESCO trial, fruquintinib was associated with favorable overall survival outcomes in previously treated patients with advanced CRC, with a median overall survival (OS) of 7.4 months and a median progression-free survival (PFS) of 3.7 months [2]. However, the objective response rate (ORR) was only 2%, and the disease control rate (DCR) was 56% [2]. Despite demonstrating clinical activity, the antitumor activity of fruquintinib monotherapy remains modest, highlighting the need for more effective therapeutic strategies [3].

Fruquintinib was originally developed and first approved in China for patients with metastatic CRC who had progressed after standard therapies. Owing to its favorable efficacy, manageable toxicity profile, and broad clinical availability, fruquintinib has become one of the most widely used later-line treatment options for advanced CRC in China. Nevertheless, despite improvements in survival, outcomes remain unsatisfactory for many patients, prompting active exploration of novel combination strategies in routine clinical practice.

In parallel, considerable research efforts in China have focused on overcoming the limited responsiveness of proficient mismatch repair (pMMR) CRC to immune checkpoint inhibitors. Based on preclinical and translational evidence suggesting that antiangiogenic therapy may improve antitumor immunity, fruquintinib combined with PD-1 inhibitors has been increasingly investigated in both prospective and retrospective studies. However, this combination remains investigational and has not been established as a standard treatment strategy in most international guidelines.

Immune checkpoint inhibitors have substantially improved outcomes for patients with mismatch repair-deficient (dMMR) CRC [4]. However, patients with pMMR CRC generally derive limited clinical benefit from PD-1/PD-L1 blockade alone [5]. To date, no randomized phase III trial has demonstrated a clear role for PD-1 inhibitors in pMMR CRC.

Fruquintinib, as a highly selective antiangiogenic agent targeting vascular endothelial growth factor receptors (VEGFR)-1, -2, and − 3, may normalize tumor vasculature and modulate the tumor microenvironment, thereby potentially enhancing antitumor immune responses [6].

Several prospective and retrospective studies have evaluated fruquintinib combined with PD-1 inhibitors in patients with advanced CRC. In a study conducted by Zhao et al., fruquintinib plus tislelizumab achieved an ORR of 20% and a DCR of 95%, with a median OS of 13.7 months and a median PFS of 9.6 months [7]. Similarly, Guo et al. reported a median OS of 17.3 months and a median PFS of 6.9 months with fruquintinib plus sintilimab, together with an ORR of 20% and a DCR of 92% [8]. However, the available evidence remains fragmented, consisting primarily of small retrospective real-world studies with heterogeneous patient populations and variable survival outcomes. Moreover, because most studies were single-arm cohorts and did not report hazard ratios or comparative survival estimates, conventional time-to-event meta-analysis was not feasible. Therefore, we conducted a reconstructed patient-level meta-analysis using published Kaplan–Meier curves to generate pooled estimates of OS and PFS, together with pooled response outcomes. Our aim was to summarize the currently available real-world evidence and provide benchmark survival estimates for future prospective controlled studies.

## Methods

### Literature search

A systematic literature search was conducted in the public databases PubMed, Web of Science, Embase, and the Cochrane Central Register of Controlled Trials (CENTRAL) from database inception to January 26, 2026. Two investigators (Y.L. and R.C.) independently performed the literature search. The search terms were “colorectal or colon or colonic or rectal or rectum”, “cancer or carcinoma or tumor or tumour or neoplasm”, “fruquintinib”, and “programmed death-1 or PD-1”. This analysis was conducted following the Preferred Reporting Items for Systematic Reviews and Meta-Analyses (PRISMA) reporting guideline for individual patient data (IPD) [9].

### Inclusion and exclusion criteria

Studies were considered eligible if they met the following criteria: patients were diagnosed with CRC; pMMR status was confirmed by immunohistochemistry (IHC); patients had previously received oxaliplatin- and irinotecan-based chemotherapy; patients were treated with fruquintinib plus a PD-1 inhibitor with or without additional local therapies; Kaplan–Meier survival curves and response outcomes were reported; and the studies were real-world retrospective studies published in English. Meeting abstracts, letters, comments, and case reports were excluded.

### Data extraction

Two investigators (Y.L. and C.F.) independently extracted the relevant information from all eligible studies. The extracted data included the first author’s name, year of publication, study region, number of enrolled patients, median age, fruquintinib dosage, types of PD-1 inhibitors, primary tumor location, reported survival outcomes, and response rates. Reconstruction of individual patient survival data was performed only for studies providing Kaplan–Meier curves with sufficient graphical quality and survival information. Studies lacking adequate OS data were excluded from the pooled OS reconstruction but remained eligible for other outcome analyses when appropriate. Any discrepancies between the investigators were resolved through discussion. Because original individual patient-level data were not available from the included studies, reconstructed individual patient-level data (reconstructed IPD) were generated from published Kaplan–Meier curves using a validated reconstruction algorithm. Therefore, this study represents a reconstructed IPD meta-analysis rather than a conventional IPD meta-analysis based on original patient-level datasets. IPD integrity assessment was limited because original patient-level datasets were not available. Instead, the integrity of the reconstructed IPD was evaluated by comparing reconstructed Kaplan–Meier curves and reconstructed median survival estimates with the corresponding published survival outcomes reported in the original studies. When available, reported numbers at risk were incorporated into the reconstruction process to improve accuracy. No major discrepancies between reconstructed and published survival outcomes were identified.

### Statistical analysis and risk assessment

OS and PFS were defined as the primary endpoints, while ORR and DCR were defined as the secondary endpoints. According to the definitions reported in the original studies, OS was measured from initiation of fruquintinib plus PD-1 inhibitor therapy to death from any cause, whereas PFS was measured from treatment initiation to disease progression or death. Published Kaplan–Meier survival curves from the retrospective studies were digitized to extract the coordinates of survival time and survival probability. IPD were subsequently reconstructed using the “IPDfromKM” package in R software based on the algorithm described by N. Liu et al. [10]. When the numbers at risk were reported in the original publications, they were incorporated into the reconstruction process to improve accuracy; otherwise, IPD were reconstructed based on the total number of patients and the extracted survival coordinates. The reconstructed IPD were then analyzed using the “survival” package to reproduce Kaplan–Meier curves and estimate survival outcomes for each individual study and for the pooled dataset. Because all included studies were single-arm cohorts, the survival analyses were descriptive in nature and were limited to the estimation of survival curves, median survival times, and survival rates at predefined time points. No comparative survival analyses, Cox proportional hazards models, or hazard ratio estimations were performed. For response outcomes, the “meta” package was applied to synthesize ORR and DCR across studies using a random-effects model. Statistical heterogeneity was assessed using Cochran’s Q test, I² statistics, and τ² estimates. To evaluate the robustness of the pooled response outcomes, leave-one-out sensitivity analyses were performed by sequentially excluding each study and recalculating the pooled estimates. The methodological quality of the included retrospective studies was independently evaluated by two investigators using the Newcastle–Ottawa Scale (NOS). The NOS assesses three domains, including selection of study participants, comparability of study groups, and ascertainment of outcomes. Studies with NOS scores of 7–9 were considered high quality, scores of 5–6 were considered moderate quality, and scores below 5 were considered low quality. Any disagreements were resolved through discussion. Publication bias was explored using funnel plots and Egger’s test, and a P value < 0.05 was considered indicative of potential publication bias. Given the limited number of included studies, the results of Egger’s test were interpreted with caution. All statistical analyses were performed using R software.

## Results

**Fig. 1:**
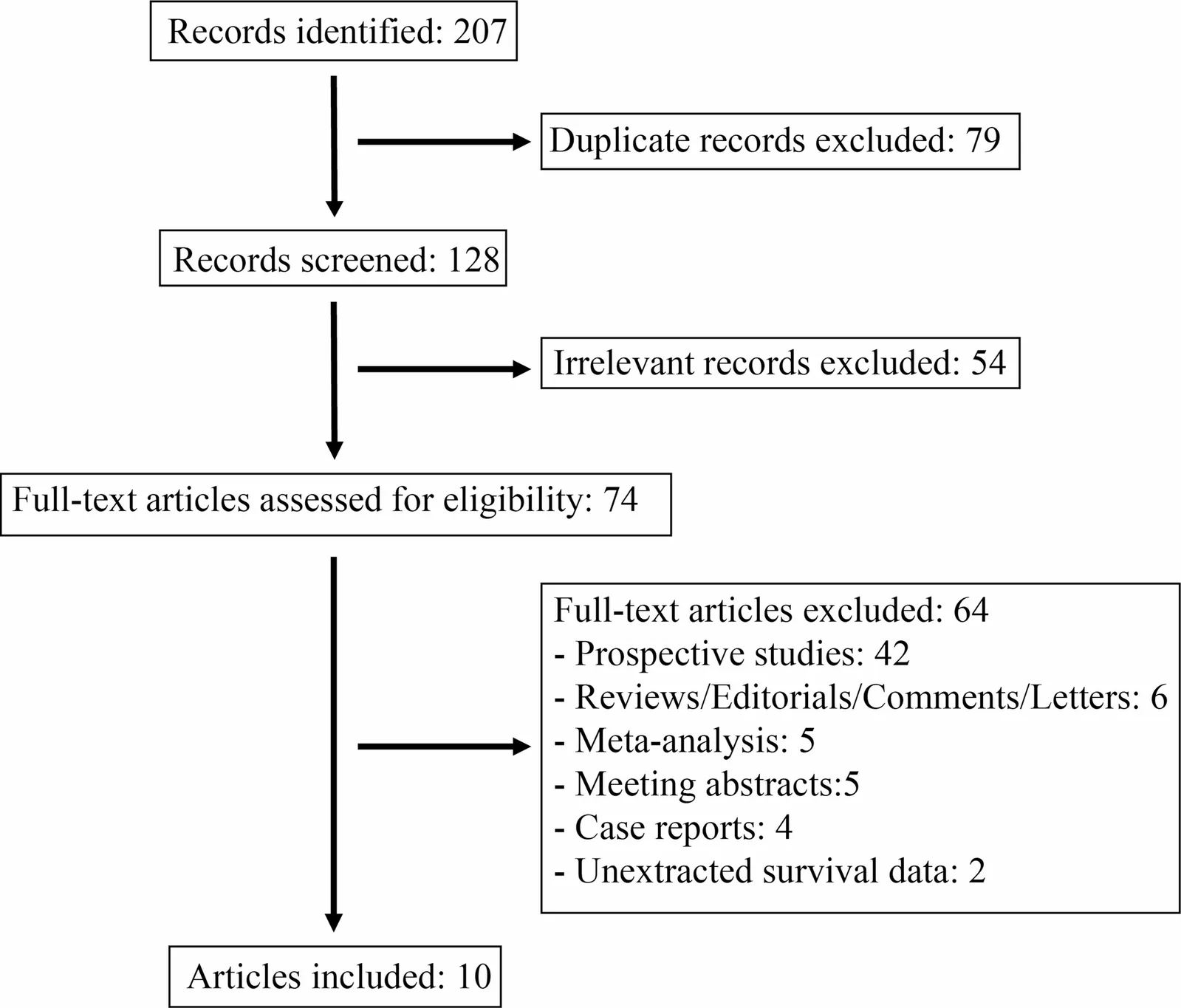
Flowchart of the study selection process for retrospective studies evaluating fruquintinib combined with PD-1 inhibitors

A total of ten real-world retrospective studies involving 609 patients with advanced CRC were identified through systematic searching and screening (Fig. 1) [11–20]. The included studies were published between 2021 and 2025, and all were conducted in mainland China. The dosage of fruquintinib ranged from 3 mg to 5 mg, with treatment cycles of either three or four weeks. The PD-1 inhibitors used across the studies included toripalimab, nivolumab, sintilimab, camrelizumab, tislelizumab, pembrolizumab, and penpulimab. The methodological quality of the included studies was generally acceptable. NOS scores ranged from 6 to 8, with all studies classified as moderate-to-high quality. Detailed quality assessment results and detailed baseline characteristics of the included studies are summarized in Table 1.

**Table 1:** Characteristics of the retrospective real-world studies evaluating fruquintinib combined with PD-1 inhibitors in patients with advanced colorectal cancer

First author	Year of publication	Region	No. patients	Age	Regimen	Immunotherapy	Left vs. Right	NOS
Liying Sun et al.	2021	Jiangxi, Chian	28	Median: 54.6 years ± 11.7	3–5 mg, d1-21, orally, every 4 weeks	toripalimab, nivolumab, sintilimab, and camrelizumab	19 vs. 9	8
Weijie Zhang et al.	2022	Henan, China	110	Median: 53 years (range: 22–81)	3–5 mg, d1-14/21, orally, every 4 weeks	sintilimab, camrelizumab, tislelizumab, pembrolizumab, and toripalimab	86 vs. 24	6
Miaomiao Gou et al.	2022	Beijing, China	45	Median: 54 years (range: 29–85)	3–5 mg, d1-21, orally, every 4 weeks	pembrolizumab, nivolumab, sintilimab, and camrelizumab	31 vs. 14	6
Xiaolin Yang et al.	2023	Hunan, China	70	Median: 59 years (range: 35–76)	3–5 mg, d1-21, orally, every 4 weeks	nivolumab, pembrolizumab, tislelizumab, sintilimab, and toripalimab	44 vs. 26	6
Luchun Li et al.	2023	Chongqing, China	47	Median: 59 years (range: 39–72)	3–4 mg, d1-21, orally, every 4 weeks	sintilimab	34 vs. 13	8
Chen Wu et al.	2024	Henan, China	29	\	\	sintilimab, camrelizumab, tislelizumab, toripalimab, nivolumab, and pembrolizumab	\	8
Kanglian Zheng et al.	2024	Beijing, China	45	Median: 58.73 years ± 7.86	3 mg, d3-21, orally, every 4 weeks	tislelizumab	37 vs. 8	8
Tianqi An et al.	2024	Henan, China	95	Median: 54 years (range: 22–81)	3–5 mg, d1-21, orally, every 4 weeks	Camrelizumab, sintilimab, tislelizumab, toripalimab, pembrolizumab, and nivolumab	73 vs. 22	8
Ying Peng et al.	2025	Jiangsu, China	63	\	3–5 mg, d1-14, orally, every 3 weeks	camrelizumab, sintilimab, tislelizumab, toripalimab, and serplulimab	49 vs. 14	8
Lina He et al.	2025	Shanghai, China	77	Median: 62 years (range: 30–80)	3–5 mg, d1-14, orally, every 3 weeks	toripalimab, nivolumab, sintilimab, camrelizumab, tislelizumab, penpulimab; and pembrolizumab	\	6

*Abbreviation*: *NOS* Newcastle–Ottawa Scale

The reported survival outcomes and response rates are presented in Table 2. Across the included studies, the median OS ranged from 14.6 to 19.5 months, while the median PFS ranged from 3.8 to 7.5 months. The ORR varied from 5.2% to 42.2%, and the DCR ranged from 55.8% to 89.3%, indicating substantial variability in reported outcomes across studies.

**Table 2:** Reported and reconstructed survival outcomes and response rates of the included retrospective real-world studies

Study	Median OS	Reconstructed OS (difference)	Median PFS	Reconstructed PFS (difference)	ORR	DCR
Liying Sun et.al.-2021 [12]	\	\	6.4 months (95% CI: 5.5–7.3)	6.41 months (+ 0.01)	7.1%	89.3%
Weijie Zhang et.al.-2022 [15]	\	\	5.4 months (95%CI: 4.0-6.8)	5.31 months (-0.09)	11.8%	70%
Miaomiao Gou et.al.-2022 [18]	14.9 months (95% CI: 7.6–21.7)	14.74 months (-0.16)	3.8 months (95% CI: 2.8–4.8)	3.81 months (+ 0.01)	11.1%	62.2%
Xiaolin Yang et.al.-2023 [16]	19.5 months (95% CI: 15.0-NA)	19.62 months (+ 0.12)	5.5 months (95% CI: 4.3–6.8)	5.46 months (-0.04)	11.4%	84.3%
Luchun Li et.al.-2023 [19]	\	\	6.0 months (95% CI: 4.6–7.4)	5.90 months (-0.10)	17.0%	80.9%
Chen Wu et.al.-2024 [20]	16.0 months (95% CI: 10.2–21.8)	16.03 months (+ 0.03)	4.2 months (95% CI: 3.2–5.2)	4.2 months (0)	6.9%	62.1%
Kanglian Zheng et.al.-2024 [13]	15.3 months (95% CI:12.6–18.0)	15.35 months (+ 0.05)	7.5 months (95% CI:5.3–9.7)	7.21 months (-0.29)	42.2%	82.2%
Tianqi An et.al.-2024 [14]	16.7 months (95%CI: 11.1–22.3)	16.86 months (+ 0.16)	4.9 months (95%CI: 3.0-6.8)	4.82 months (-0.08)	11.6%	74.7%
Ying Peng et.al.-2025 [11]	\	\	7.0 months	6.54 months (-0.46)	7.9%	74.6%
Lina He et.al.-2025 [17]	14.6 months (95% CI: 9.6–15.6)	13.69 months (-0.91)	5.1 months (95% CI: 3.6–6.7)	4.48 months (-0.62)	5.2%	55.8%

**Fig. 2:**
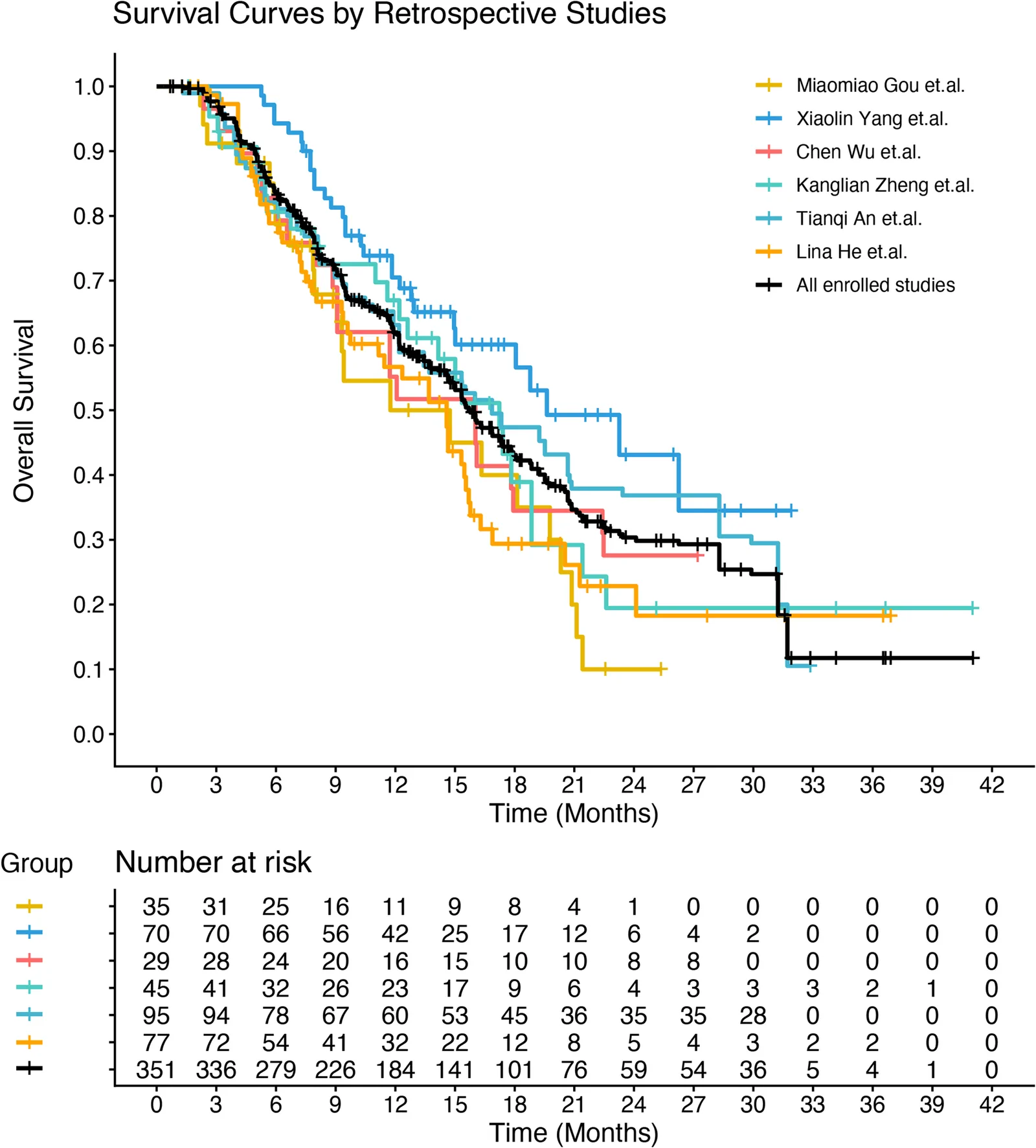
Kaplan–Meier curve for pooled overall survival based on reconstructed individual patient-level data (reconstructed IPD) derived from six retrospective real-world studies that provided sufficient Kaplan–Meier information for survival reconstruction

**Fig. 3:**
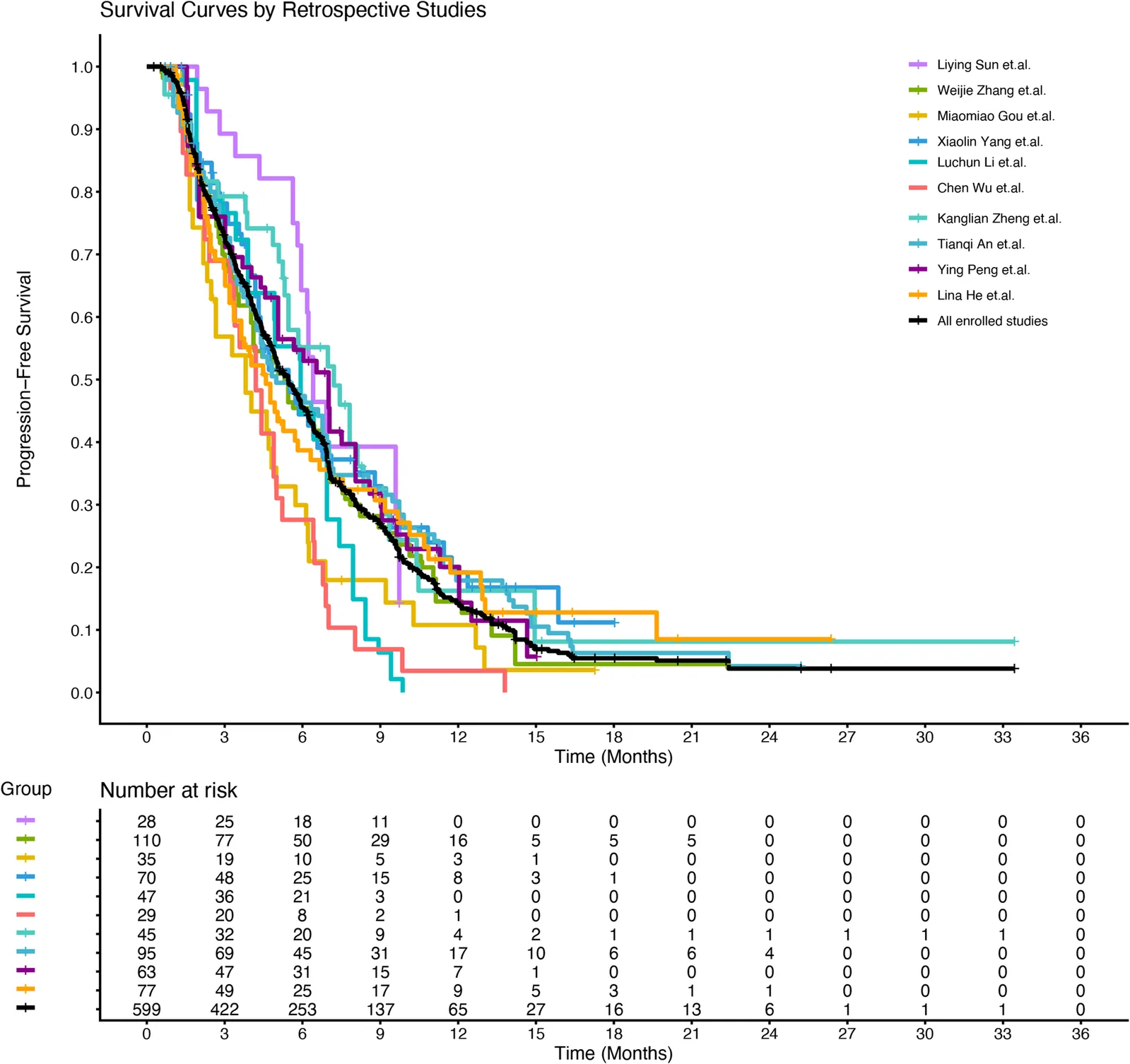
Kaplan–Meier curve for pooled progression-free survival based on reconstructed individual patient-level data (reconstructed IPD) derived from ten retrospective real-world studies

Among the 10 included studies, reconstructed OS analyses were feasible for six studies that provided sufficient Kaplan–Meier data, whereas PFS analyses were feasible for all ten studies. ORR and DCR analyses included all eligible studies reporting these outcomes. Based on reconstructed patient-level data, the pooled median OS was 15.7 months, and the pooled median PFS was 5.5 months (Figs. 2 and 3). The estimated OS rates at 6, 9, and 12 months were 83.5% (95% CI, 79.6–87.6), 71.8% (95% CI, 67.1–76.8), and 62.0% (95% CI, 56.9–67.6), respectively. Correspondingly, the PFS rates at 6, 9, and 12 months were 45.6% (95% CI, 41.7–49.8), 27.0% (95% CI, 23.5–31.0), and 14.3% (95% CI, 11.6–17.7), respectively (Table 3).

**Table 3:** Reported efficacy outcomes of retrospective studies evaluating fruquintinib combined with PD-1 inhibitors in advanced colorectal cancer

Survival rates	6 months	9 months	12 months
OS	83.5% (95% CI 79.6–87.6)	71.8% (95% CI 67.1–76.8)	62.0% (95% CI 56.9–67.6)
PFS	45.6% (95% CI 41.7–49.8)	27.0% (95% CI 23.5–31.0)	14.3% (95%CI 11.6–17.7)

To assess the integrity and accuracy of the reconstructed IPD, reconstructed median survival estimates were compared with the corresponding values reported in the original publications (Table 2). Among studies with available survival data, the absolute differences between reported and reconstructed median OS ranged from 0.03 to 0.91 months, whereas the differences for median PFS ranged from 0 to 0.62 months. Overall, reconstructed survival estimates closely approximated the published results, supporting the validity and reliability of the reconstructed IPD for subsequent pooled survival analyses.

**Fig. 4:**
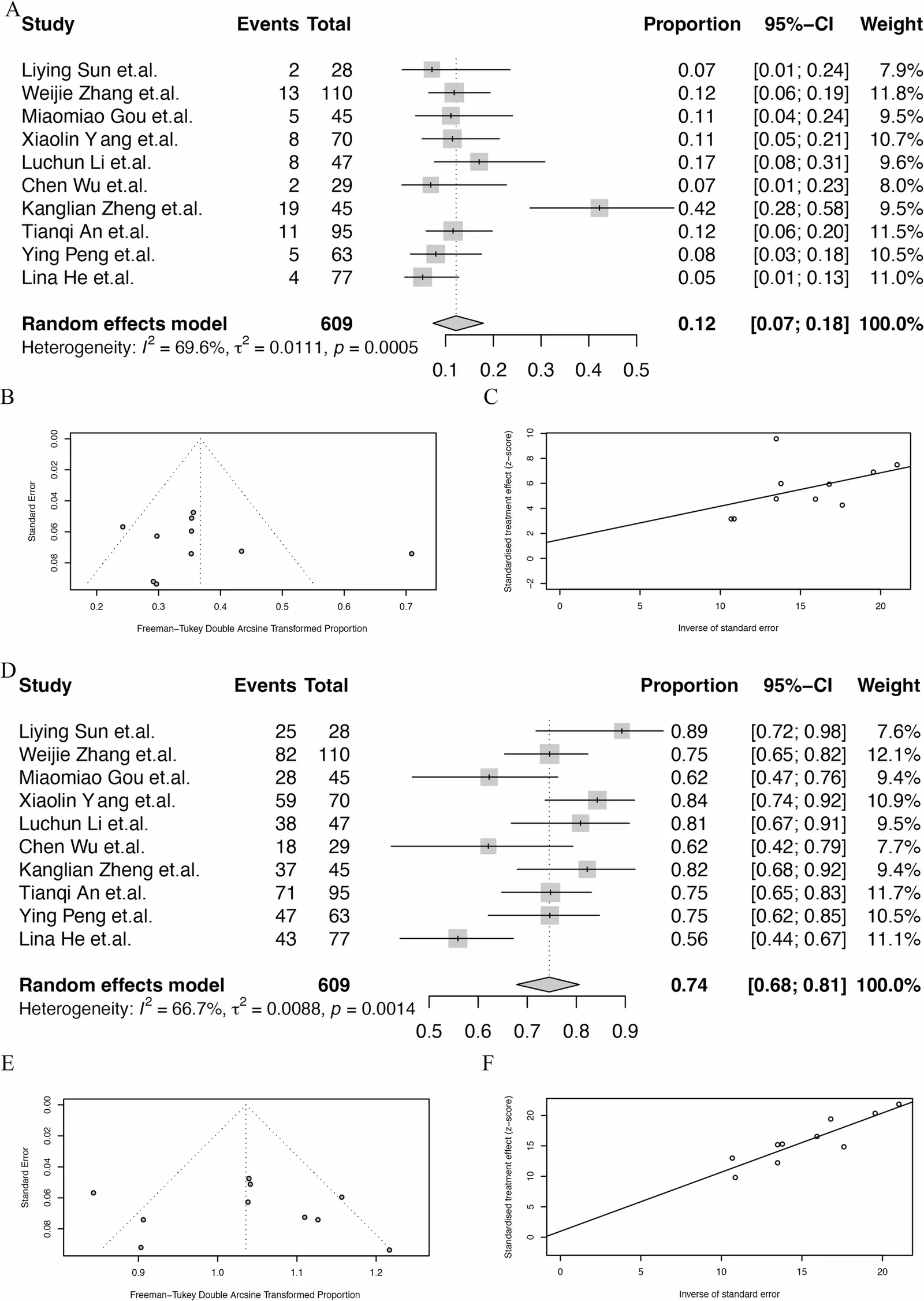
Pooled single-arm response rates across the included retrospective studies. **A** Pooled objective response rate. **B** and **C** Funnel plot and Egger’s test for publication bias of the objective response rate. **D** Pooled disease control rate. **E** and **F** Funnel plot and Egger’s test for publication bias of the disease control rate

Response data were available for all included studies. The pooled ORR was 12% (95% CI, 7–18), with substantial between-study heterogeneity (I² = 69.6%, τ² = 0.0111, p for heterogeneity = 0.0005). Similarly, the pooled DCR was 74% (95% CI, 68–81), with substantial heterogeneity across studies (I² = 66.7%, τ² = 0.0088, p for heterogeneity = 0.0014) (Fig. 4A and D). Funnel plot inspection, together with Egger’s tests (*p* = 0.6113 for ORR and *p* = 0.7313 for DCR), did not reveal any significant evidence of publication bias (Fig. 4B, C and E, and 4F). However, given the limited number of included studies, the statistical power of these analyses was limited. Leave-one-out sensitivity analyses were conducted to evaluate the robustness of the pooled response outcomes. Sequential omission of individual studies yielded pooled ORR estimates ranging from 9.9% to 13.2%, compared with 12.2% in the primary analysis. Similarly, pooled DCR estimates ranged from 73.1% to 76.6%, compared with 74.5% in the primary analysis. No single study materially influenced the pooled effect estimates, supporting the robustness of the results despite the observed between-study heterogeneity (Table 4).

**Table 4:** Leave-one-out sensitivity analyses for pooled objective response rate (ORR) and disease control rate (DCR)

ORR					
	proportion	95%-CI	tau^2	tau	I^2
Omitting Liying Sun et.al.-2021 [12]	0.1269	[0.0742; 0.1902]	0.0124	0.1112	72.50%
Omitting Weijie Zhang et.al.-2022 [15]	0.1227	[0.0680; 0.1893]	0.0135	0.1162	72.90%
Omitting Miaomiao Gou et.al.-2022 [18]	0.1232	[0.0697; 0.1882]	0.0131	0.1144	72.90%
Omitting Xiaolin Yang et.al.-2023 [16]	0.1230	[0.0688; 0.1889]	0.0133	0.1154	72.90%
Omitting Luchun Li et.al.-2023 [19]	0.1172	[0.0657; 0.1800]	0.0125	0.1118	71.90%
Omitting Chen Wu et.al.-2024 [20]	0.1272	[0.0745; 0.1906]	0.0123	0.1109	72.40%
Omitting Kanglian Zheng et.al.- 2024 [13]	0.0994	[0.0749; 0.1266]	0	0	0%
Omitting Tianqi An et.al.-2024 [14]	0.1230	[0.0684; 0.1894]	0.0134	0.1160	72.90%
Omitting Ying Peng et.al.-2025 [11]	0.1275	[0.0736; 0.1925]	0.0125	0.1119	71.90%
Omitting Lina He et.al.-2025 [17]	0.1324	[0.0803; 0.1941]	0.0105	0.1026	67.40%
Random effects model	0.1219	[0.0737; 0.1793]	0.0111	0.1052	69.60%
DCR					
Omitting Liying Sun et.al.-2021 [12]	0.7310	[0.6647; 0.7928]	0.0075	0.0868	65.10%
Omitting Weijie Zhang et.al.-2022 [15]	0.7449	[0.6684; 0.8147]	0.0108	0.1041	70.40%
Omitting Miaomiao Gou et.al.-2022 [18]	0.7564	[0.6886; 0.8187]	0.0084	0.0916	66.50%
Omitting Xiaolin Yang et.al.-2023 [16]	0.7314	[0.6623; 0.7955]	0.0080	0.0892	63.70%
Omitting Luchun Li et.al.-2023 [19]	0.7378	[0.6649; 0.8049]	0.0097	0.0984	68.90%
Omitting Chen Wu et.al.-2024 [20]	0.7542	[0.6859; 0.8169]	0.0088	0.0936	67.90%
Omitting Kanglian Zheng et.al.- 2024 [13]	0.7363	[0.6643; 0.8026]	0.0093	0.0965	68.30%
Omitting Tianqi An et.al.-2024 [14]	0.7446	[0.6684; 0.8143]	0.0108	0.1038	70.30%
Omitting Ying Peng et.al.-2025 [11]	0.7448	[0.6696; 0.8136]	0.0106	0.1030	70.40%
Omitting Lina He et.al.-2025 [17]	0.7662	[0.7155; 0.8135]	0.0029	0.0541	44.30%
Random effects model	0.7447	[0.6784; 0.8060]	0.0088	0.0936	66.70%

## Discussion

In this reconstructed patient-level analysis of real-world retrospective studies, patients with advanced pMMR CRC treated with fruquintinib plus PD-1 inhibitors achieved a pooled median OS of 15.7 months and a median PFS of 5.5 months. Although the pooled DCR was relatively high, the ORR remained modest at 12%, with substantial variability across studies. This discrepancy between disease stabilization and tumor shrinkage highlights the complexity of interpreting clinical activity in this setting and underscores the need for comparative studies to determine the specific contribution of PD-1 inhibitors within fruquintinib-based treatment strategies. The pooled median OS observed in the present analysis appears substantially longer than that reported in the pivotal FRESCO trial. However, this difference should not be interpreted as direct evidence of a survival advantage attributable to the addition of PD-1 inhibitors. Several factors may account for the observed discrepancy. First, the included studies were retrospective real-world cohorts and may have been subject to patient selection bias. Second, baseline prognostic characteristics differed across studies and were incompletely reported, limiting direct comparisons. For example, a relatively high proportion of patients had left-sided primary tumors, which are generally associated with more favorable outcomes in metastatic colorectal cancer. Third, variations in prior lines of therapy, performance status, metastatic burden, and the use of local treatments such as radiotherapy may have influenced survival outcomes. Therefore, cross-study comparisons with historical fruquintinib monotherapy trials should be interpreted cautiously. The substantially longer OS observed in the present analysis compared with historical fruquintinib monotherapy trials should be interpreted cautiously, as differences in patient selection and baseline prognostic characteristics may have contributed to the observed outcomes.

In contemporary oncology practice, combining immunotherapy with antiangiogenic agents has become a widely explored strategy to enhance treatment efficacy [21]. However, evidence from other malignancies suggests that such combinations do not consistently yield superior outcomes. For example, in hepatocellular carcinoma, lenvatinib monotherapy demonstrated efficacy comparable to bevacizumab plus atezolizumab [22]. Furthermore, the addition of pembrolizumab to lenvatinib failed to significantly improve survival outcomes in the LEAP-002 trial, and dual immunotherapy did not outperform standard therapies in the CheckMate 9DW trial [23, 24]. To date, no randomized clinical trials have demonstrated the superiority of combining PD-1 inhibitors with fruquintinib in pMMR CRC. Therefore, the incremental benefit of this combination remains uncertain and requires further validation.

Whether immunotherapy may have a greater role earlier in the treatment course of pMMR CRC remains an area of active investigation. Emerging studies have suggested that multimodal strategies incorporating immunotherapy and radiotherapy may improve treatment responses in selected settings [25–27]. However, the relevance of these findings to patients receiving later-line fruquintinib-based therapy remains uncertain and requires further prospective evaluation. These findings highlight the potential importance of multimodal treatment strategies and underscore the need for well-designed randomized trials to define the role of PD-1 inhibitors in this setting.

In real-world clinical practice, combination strategies often extend beyond systemic therapy. Local treatments, particularly in patients with oligometastatic disease, may contribute substantially to improved outcomes [28]. However, the relative contribution of these interventions remains unclear and may confound the interpretation of clinical activity attributed to immunotherapy.

Substantial heterogeneity was observed across the included studies. Potential contributors include differences in fruquintinib dosing schedules, selection of PD-1 inhibitors, incorporation of local therapies, baseline patient characteristics, and prior treatment histories. In addition, several studies enrolled highly selected patient populations, which may have further contributed to variability in treatment outcomes. Consistent with these observations, considerable statistical heterogeneity was identified in the pooled response analyses (ORR: I² = 69.6%; DCR: I² = 66.7%). Although leave-one-out sensitivity analyses suggested that the pooled estimates were generally robust and were not driven by any single study, the underlying sources of heterogeneity could not be formally explored because of limited and inconsistently reported study-level data. Therefore, the pooled efficacy outcomes should be interpreted with appropriate caution.

An additional unresolved issue is the optimal sequencing of immunotherapy. It remains unclear whether PD-1 inhibitors should be continued beyond disease progression. Evidence from studies such as CheckMate 9DW suggests that immunotherapy may confer delayed or sustained benefits in certain contexts [24]. In our clinical practice, immunotherapy is typically discontinued upon progression during combination treatment; however, the potential value of treatment beyond progression warrants further investigation.

Tumor heterogeneity may also influence treatment outcomes. In the included studies, a higher proportion of tumors originated from the left-sided colon; however, detailed survival analyses stratified by tumor location were not available. Notably, previous studies have demonstrated that primary tumor sidedness is an important prognostic factor in metastatic colorectal cancer, with left-sided tumors generally associated with more favorable clinical outcomes. The pivotal phase III FRESCO trial also included a substantial proportion of patients with left-sided primary tumors. Nevertheless, differences in the distribution of baseline characteristics, including tumor sidedness and prior treatment exposure, may complicate cross-study comparisons between our pooled analysis and historical studies of fruquintinib monotherapy. Given that primary tumor sidedness is an established prognostic and predictive factor in metastatic colorectal cancer and has been associated with differential responses to targeted therapies, it is plausible that it may also influence outcomes with antiangiogenic and immunotherapy-based combinations. Further studies are warranted to clarify this relationship [29, 30].

Importantly, the present study adds to the existing literature by providing a quantitative synthesis of survival outcomes derived from reconstructed patient-level data across multiple real-world studies. Although individual retrospective reports have described the efficacy of fruquintinib combined with PD-1 inhibitors, the reported survival outcomes have varied considerably, making interpretation challenging. By reconstructing individual patient survival data from published Kaplan–Meier curves, we were able to generate pooled estimates of OS and PFS and to summarize the reported clinical outcomes associated with this treatment strategy in routine clinical practice. These results may serve as a benchmark for the design and interpretation of future prospective controlled studies evaluating fruquintinib-based combination therapies in advanced colorectal cancer.

The present analysis provides a comprehensive summary of real-world outcomes associated with fruquintinib combined with PD-1 inhibitors in advanced pMMR colorectal cancer. The pooled survival estimates provide a descriptive summary of outcomes observed across retrospective real-world studies. Because all included studies were single-arm cohorts without comparator groups, no conclusions can be drawn regarding the comparative efficacy or incremental contribution of PD-1 inhibitors to fruquintinib-based therapy. Therefore, the findings should be interpreted as hypothesis-generating rather than practice-changing. The primary contribution of this study is to provide benchmark survival data that may inform the design, conduct, and interpretation of future prospective controlled trials.

Several limitations of this analysis should be acknowledged. First, duration of response (DOR) data were not consistently reported across the included studies, limiting the assessment of response durability. Second, although individual patient data were reconstructed from published Kaplan–Meier curves using a validated methodology, reconstructed datasets cannot fully substitute for original patient-level data. Potential inaccuracies may arise from curve digitization, censoring estimation, and reconstruction procedures. In addition, the reconstruction process relies on estimated censoring patterns derived from published Kaplan–Meier curves. For studies lacking complete numbers-at-risk tables, censoring distributions could only be inferred algorithmically, which may introduce additional uncertainty into the reconstructed survival estimates. While quantitative validation demonstrated close agreement between reconstructed and originally reported survival outcomes, certain patient-level variables, treatment details, and potential confounding factors could not be verified or adjusted for. Moreover, because original individual patient data and hazard ratio estimates were largely unavailable, conventional pooled time-to-event meta-analyses and adjusted survival analyses could not be reliably performed. In addition, reconstructed OS analyses were feasible for only six studies because adequate Kaplan–Meier information was unavailable for the remaining studies. Consequently, exclusion of these studies from the pooled OS reconstruction may have introduced selection bias and should be considered when interpreting the pooled survival estimates. Third, considerable clinical heterogeneity existed across the included studies with respect to fruquintinib dosing schedules, PD-1 inhibitor selection, concomitant local treatments, and baseline patient characteristics. However, detailed study-level and patient-level information was not consistently available, precluding formal subgroup analyses, exploration of heterogeneity sources, and adjustment for baseline confounders. Fourth, all included studies were single-arm retrospective cohorts and no comparator group was available. Consequently, the present analysis cannot determine whether the addition of PD-1 inhibitors improves survival or response outcomes compared with fruquintinib monotherapy or other treatment strategies. Although pooled survival estimates were generated, these results should be interpreted as descriptive observations rather than evidence of comparative treatment effects. Any comparisons with historical studies are inherently subject to bias arising from differences in patient populations, baseline characteristics, treatment exposure, and study design. Fifth, all included studies were retrospective real-world studies conducted in China, which may limit the generalizability of our findings to other populations. Furthermore, assessment of publication bias was limited by the relatively small number of included studies. Although funnel plots and Egger’s tests did not detect statistical evidence of publication bias, the power of these methods was limited. Finally, safety outcomes were not systematically analyzed because adverse event reporting was inconsistent and incomplete across the included studies. Information regarding grade ≥ 3 adverse events, treatment discontinuation, dose reductions, and immune-related toxicities was frequently unavailable. Consequently, the potential toxicity associated with combination and multimodal therapies may have been underestimated, and the overall risk–benefit profile of this treatment strategy remains uncertain.

## Conclusion

In conclusion, this reconstructed patient-level analysis describes real-world outcomes of fruquintinib combined with PD-1 inhibitors in patients with advanced pMMR colorectal cancer. The pooled survival estimates provide a descriptive summary of outcomes observed in retrospective clinical practice. However, given the retrospective nature of the included studies, substantial heterogeneity across cohorts, and the absence of any direct or indirect controlled comparison, the current evidence cannot determine whether the addition of PD-1 inhibitors influences clinical outcomes beyond those expected with fruquintinib-based therapy alone. Therefore, the results should be interpreted strictly as hypothesis-generating. While these data may be useful as a benchmark for future research, they do not support any inference of treatment superiority or clinical advantage over fruquintinib monotherapy or other standard regimens. Prospective randomized controlled trials are required to determine whether adding PD-1 blockade to fruquintinib alters clinical outcomes and to identify patient subgroups that may derive differential outcomes.

## Supplementary Information

Supplementary Material 1.

## Data Availability

All enrolled prospective studies can be searched and downloaded from official websites. DOI numbers:1. https://doi.org/10.3389/fonc.2021.754881;2. ﻿https://doi.org/10.3389/fonc.2022.929342;3. https://doi.org/10.3389/fonc.2022.851756;4. https://doi.org/10.21037/jgo-23-931;5. https://doi.org/10.21037/tcr-23-867;6. ﻿https://doi.org/10.3389/fonc.2024.1480704;7. https://doi.org/10.3389/fonc.2024.1420956;8. ﻿https://doi.org/10.4251/wjgo.v16.i6.2449;9. ﻿https://doi.org/10.3892/ol.2025.15287 ;10. https://doi.org/10.1016/j.clon.2024.103700.
